# "The Failing Watchman": Recurrent Left Atrial Appendage Occlusion Device (LAAOD) Thrombus After Successful Initial Implantation

**DOI:** 10.7759/cureus.61368

**Published:** 2024-05-30

**Authors:** Omar Khan, Antoine Egbe, Danish Abbasi

**Affiliations:** 1 Internal Medicine, McLaren Macomb Hospital, Mount Clemens, USA; 2 Internal Medicine, Arkansas College of Osteopathic Medicine, Fort Smith, USA; 3 Internal Medicine, Beaumont Hospital, Dearborn, USA; 4 Cardiovascular Diseases, University of Arkansas, Little Rock, USA

**Keywords:** device-associated thrombus, anticoagulation protocol, recurrent thrombotic event, watchman device, direct oral anticoagulants, left atrial appendage closure

## Abstract

A 79-year-old female with chronic atrial fibrillation was being treated with dabigatran (Pradaxa). Pradaxa was discontinued after a significant bleeding episode. A WATCHMAN device was successfully implanted and Pradaxa was started. A transesophageal echocardiogram (TEE) 49 days later showed a 3.6×2.2 cm clot overlying the device. Pradaxa was switched to Coumadin. Subsequent TEEs showed the complete resolution of the thrombus after five months on Coumadin. Coumadin was discontinued. Six months later, TEE showed a large mobile thrombus attached to the left atrial appendage occlusion device (LAAOD). The patient's hypercoagulable workup was negative. Due to recurrent thrombotic events, she was started on apixaban (Eliquis) due to a prior history of bleeding on Coumadin. She is currently on Eliquis with no further episodes of bleeding or device thrombus.

## Introduction

Left atrial appendage (LAA) closure using an occlusive device is increasingly emerging as an alternative to anticoagulation (AC) for stroke prevention in atrial fibrillation (AF) patients with a high risk of bleeding. Device-associated thrombus (DAT) is a known complication, which resolves with treatment [1]. We present a very rare case of a patient suffering from recurrent DAT after the initial successful implantation of a WATCHMAN device.

## Case presentation

A 79-year-old female with a history of hypertension, hypothyroidism, and chronic AF was being treated with Coumadin. She underwent a sigmoidectomy with a Hartmann stump and diverting colostomy one year later due to her complicated diverticulitis. Successful reversal of the colostomy occurred within that same year, with a brief interruption of AC during both surgeries, but without any thromboembolic events. Subsequently, AC was switched to dabigatran (Pradaxa). Six years later, the patient was hospitalized for significant lower gastrointestinal bleeding (LGIB) and a decrease in hemoglobin from 13 to 10 g/dL. A colonoscopy revealed a colonic ulcer with hemorrhage at the colo-colonic anastomosis from the prior surgery, which was successfully treated with epinephrine injection and clipping. Due to the increased risk of recurrent bleeding, it was decided to discontinue dabigatran. The patient was referred for an LAA occlusion device (LAAOD).

The patient was evaluated and deemed a suitable candidate for LAAOD insertion via right femoral venous access. A WATCHMAN access system was advanced over a guidewire through the interatrial septum into the left atrium. Through a delivery catheter and under fluoroscopic guidance, a 33 mm device was deployed/implanted into the LAA. A "tug test" was then performed to ensure proper positioning of the device, after which the device was released and the delivery system removed. Subsequently, an angiogram with contrast dye confirmed the correct placement of the device without any leakage. Post-implant transesophageal echocardiography showed the LAAOD well seated in the LAA, a normal systolic function of 55-60%, and no residual flow around the device. Pradaxa 75 mg twice daily was initiated after the device implantation.

**Figure 1:**
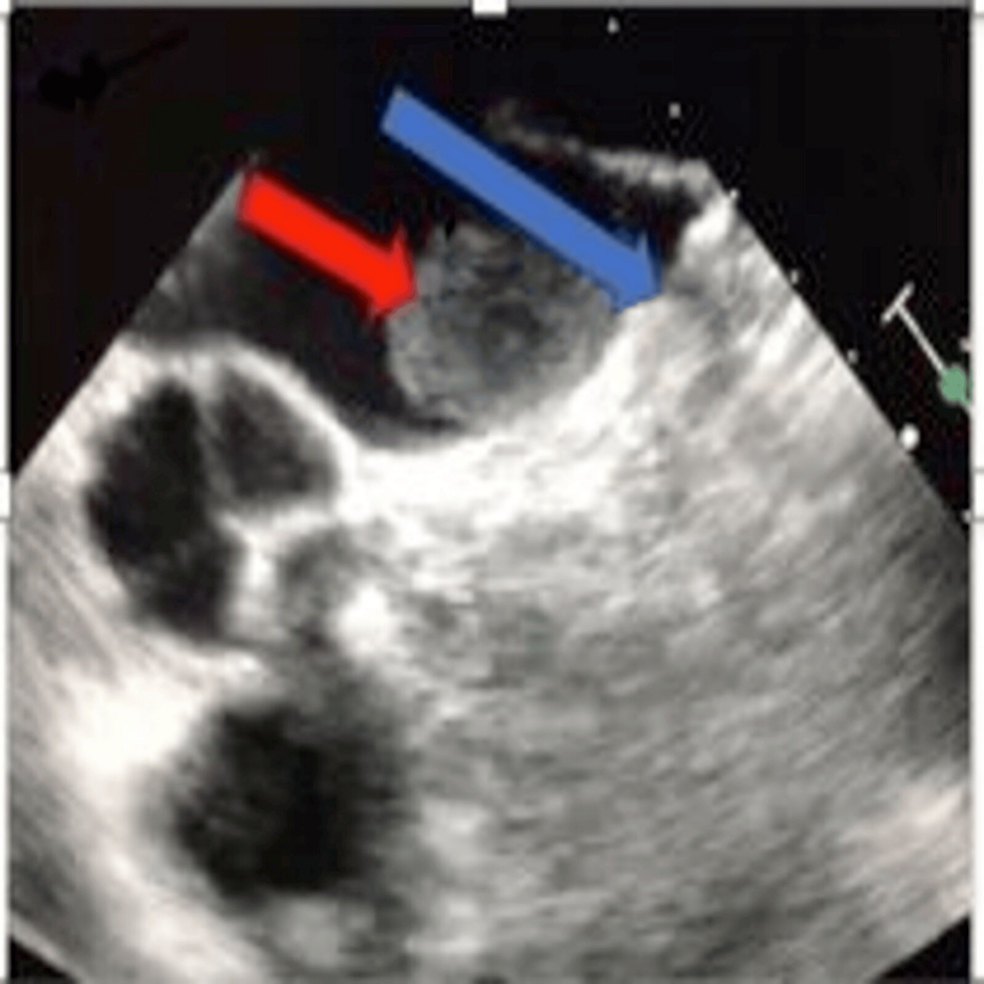
Mid-esophageal AV view of a thrombus formation as seen on TEE 49 days post-implantation of the WATCHMAN device. The red arrow indicates a thrombus, and the blue arrow points to the WATCHMAN device. AV: aortic valve; TEE: transesophageal echocardiogram

**Figure 2:**
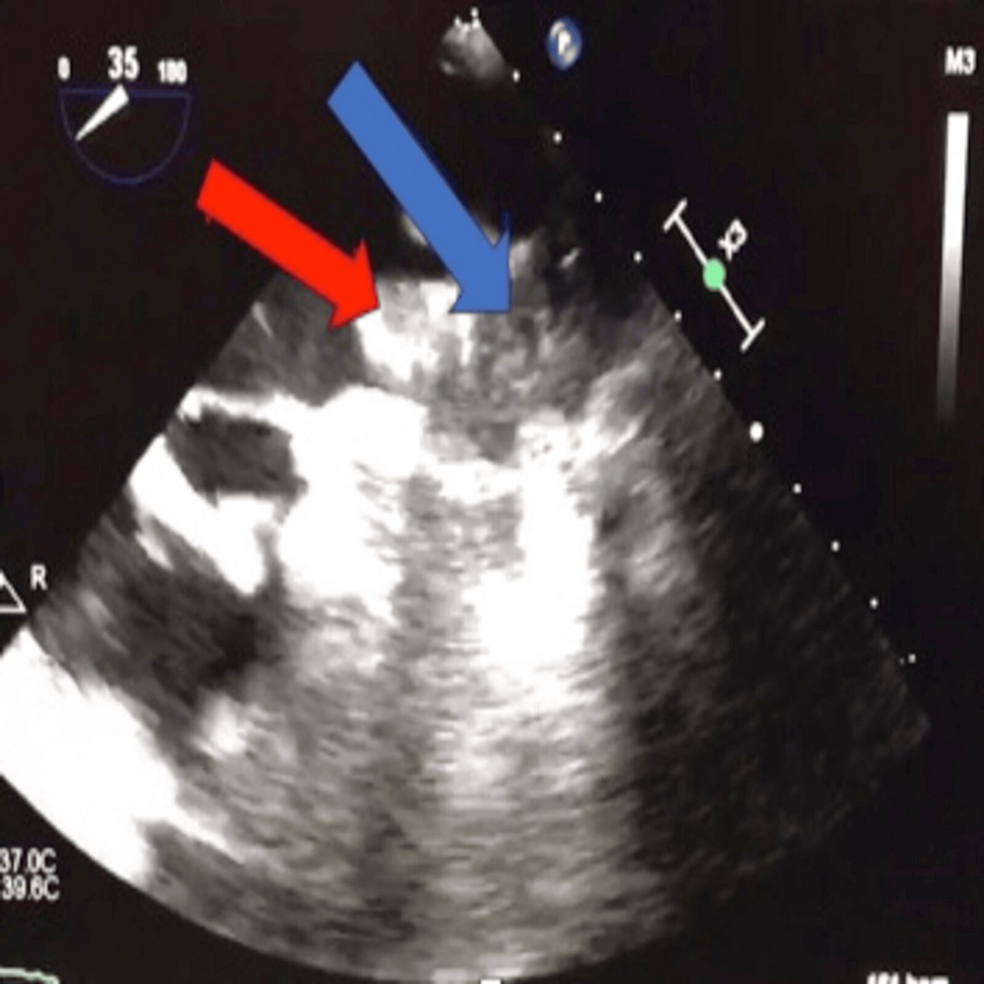
Reduction in thrombus size after 30 days of anticoagulation therapy with Coumadin. The red arrow highlights the thrombus, and the blue arrow shows the WATCHMAN device.

**Figure 3:**
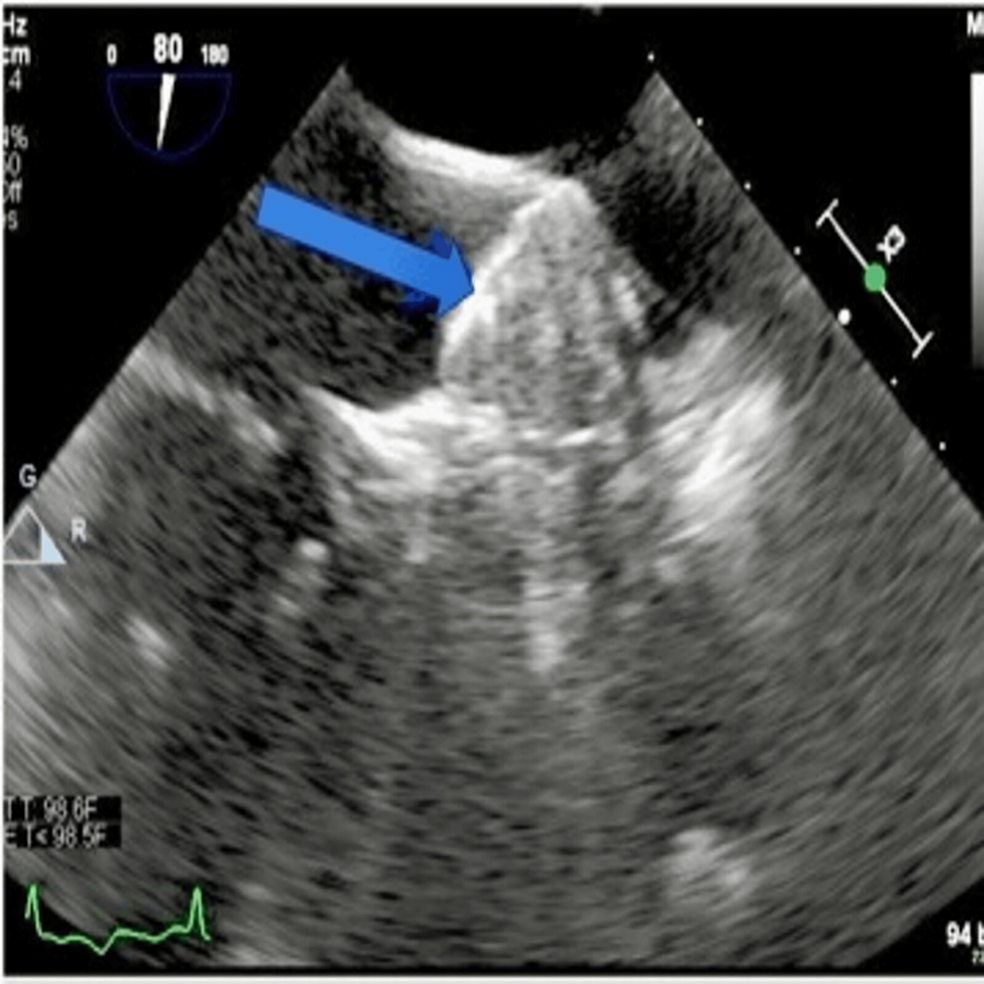
Complete resolution of thrombus observed six months post-intervention. The blue arrow identifies the WATCHMAN device.

A follow-up transesophageal echocardiogram (TEE), conducted 49 days later as per protocol, revealed a 3.6×2.2 cm clot overlying the WATCHMAN device and minimal residual flow (2 mm) around the device (Figure 1). Pradaxa was discontinued, and Coumadin bridged with unfractionated heparin was initiated. The patient was discharged on Coumadin once her international normalized ratio (INR) was therapeutic. A repeat TEE one month later showed a smaller persistent device thrombus (1.3×2 cm) compared with the previous TEE and minimal residual flow around the device (Figure 2). The patient's Coumadin treatment was maintained, and three months later, a TEE showed the thrombus had almost entirely resolved except for a tiny thrombus over the device, with persistent minimal flow (1-2 mm) around the device. Her Coumadin therapy was then extended for an additional three weeks. One month following the cessation of Coumadin, a follow-up TEE showed no evidence of thrombus on the device (Figure 3).

The patient was admitted to the hospital six months later with acute respiratory distress due to a massive pulmonary embolism (PE) and cor pulmonale requiring mechanical ventilation. She underwent emergent pulmonary embolectomy and had an inferior vena cava (IVC) filter placed. Initially, she was treated with unfractionated heparin, but then was subsequently discharged to a long-term acute care hospital (LTACH) after an extended stay in the ICU. Her hospitalization was further complicated by acute renal failure (ARF), requiring dialysis. She was discharged without oral AC due to her history of significant LGIB and recent evidence of heme-positive stool and anemia. However, she was successfully weaned off ventilation, her ARF resolved to which she no longer required dialysis. After a one-month stay in LTACH, she was eventually transferred to a rehabilitation facility.

**Figure 4:**
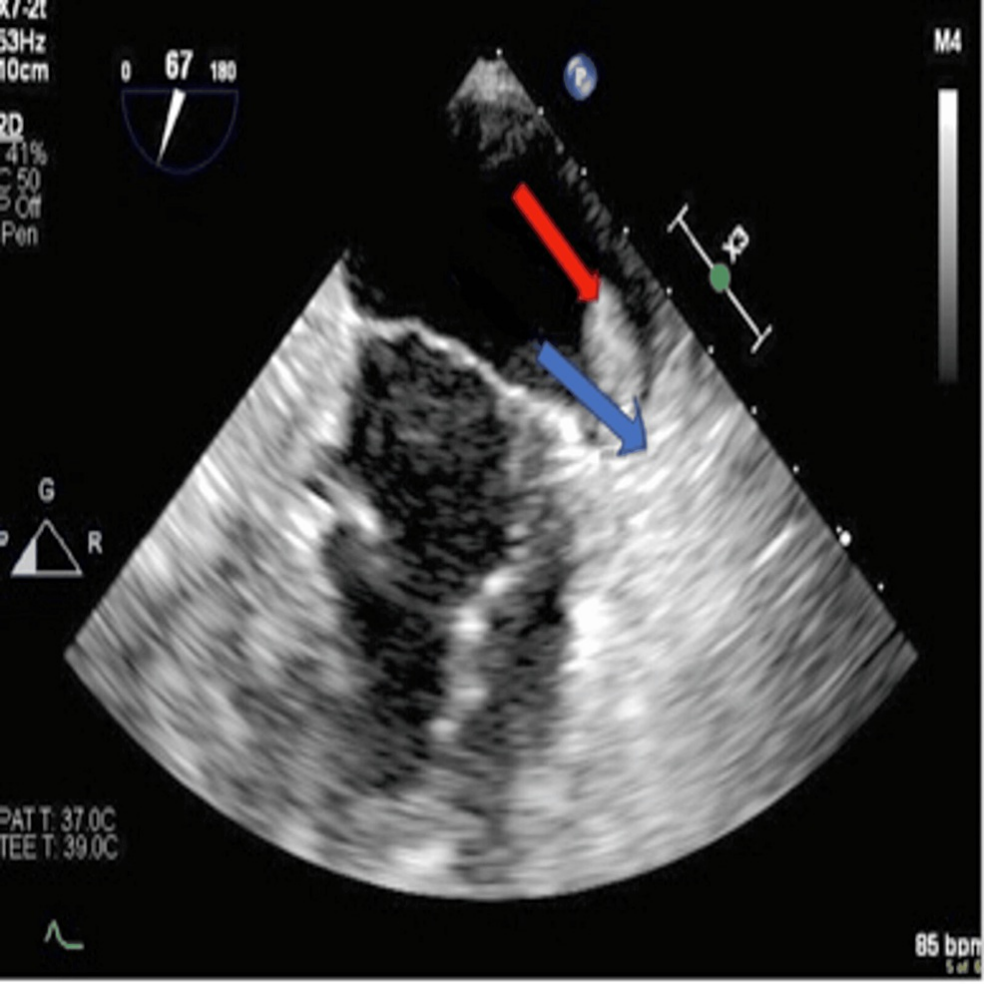
Recurrence of thrombus on the WATCHMAN device, observed eight months following initial resolution. The red arrow indicates a thrombus, and the blue arrow points to the WATCHMAN device.

Two months later, the patient was readmitted to the hospital with pneumonia. Owing to *Staphylococcus* bacteremia, a TEE was performed, revealing no vegetation but a large, mobile thrombus attached to the LAAOD as seen in Figure 4. Given her recurrent thrombotic events over the past year and following consultation with the hematology team, she was started on apixaban (Eliquis), as the benefits of anticoagulation therapy clearly outweighed the risks at this juncture. A hypercoagulable state workup was conducted, yielding negative results for protein C/S and antithrombin deficiencies, factor V Leiden, and prothrombin gene mutation. She was discharged on Eliquis and has experienced no further complications.

## Discussion

Direct oral anticoagulants (DOACs) and direct inhibitors of thrombin or factor Xa (FXa) have emerged as alternatives to warfarin for the prevention of stroke in patients with nonvalvular atrial fibrillation (NVAF), but like any oral anticoagulant, their use is associated with an increased risk of bleeding events. Studies have found the LAA to be the source of thrombus in the majority of cases of thromboembolism associated with AF. Blackshear and Odell reviewed 23 separate studies including 4,792 patients with AF evaluating the etiology of thrombus by transesophageal echocardiography, autopsy, and operation. It was found that thrombi were localized to or were present in the LAA in 254 of 446 (57%) patients with rheumatic AF and 201 of 222 (91%) patients with nonrheumatic AF [1].

LAA closure has emerged as a reasonable alternative therapy to oral anticoagulation (OAC) for stroke prevention in patients with AF and a CHA2DS2-VASc score of ≥1. The PROTECT AF was a prospective randomized trial comparing outcomes between LAA occlusion with a WATCHMAN device and warfarin. In it, 707 patients with NVAF and CHA2DS2-VASc score of ≥1 were randomly assigned in a 2:1 ratio to undergo LAA occlusion or receive chronic warfarin therapy, respectively. Patients in the WATCHMAN arm received concomitant warfarin and aspirin for 45 days to facilitate device endothelialization. The main outcomes measured were a composite of stroke, systemic embolism, and cardiovascular/unexplained death. There were 39 events among the 463 patients (8.4%) in the device group compared with 34 events among 244 patients (13.9%) in the warfarin group after a mean follow-up of 3.8 years. LAA occlusion met the criteria for both non-inferiority and superiority compared to warfarin for preventing stroke, systemic thromboembolism, and cardiovascular death, with a significant reduction in bleeding (4.8% versus 7.4%) [2]. 

Similarly, in the PREVAIL trial, 407 patients were randomized in a 2:1 ratio to undergo LAA occlusion with a WATCHMAN device and subsequent discontinuation of warfarin (n=269) or receive chronic warfarin therapy (n=138). LAA occlusion was non-inferior to warfarin for the prevention of ischemic stroke or systemic embolism >7 days post-device implantation (2.5% versus 2%) [3]. The five-year combined outcome of the PREVAIL and PROTECT AF trials also showed that the WATCHMAN device was comparable to warfarin in stroke and systemic embolism prevention in NVAF >7 days after randomization (2.5% in the device group versus 1.35% in the warfarin group), with significant less hemorrhagic stroke (0.17% versus 0.87%; p=0.0022) and non-procedure-related major bleeding (1.7% versus 3.6%; p=0.0003) with WATCHMAN device compared to warfarin [4].

On the contrary, as with all procedures, there are known complications associated with the WATCHMAN device implant. Reddy et al. reviewed the safety profile of the WATCHMAN device in a long-term follow-up of the PROTECT AF study and the Continued Access Protocol (CAP) Registry, a nonrandomized registry that began at the conclusion of the PROTECT AF trial. Some of the complications include procedure-related stroke (0.9% and 0%), device embolization (0.6% and 0%), pericardial effusion (5.8% and 2.2%), and bleeding (0.7% and 0.65%) in the PROTECT AF and in CAP Registry, respectively. The device thrombus rate was 4.2% in the PROTECT AF. The rate of serious pericardial effusion within seven days of implantation was lower in the CAP Registry (5.0% versus 2.2%), demonstrating that, as with other procedures, complications associated with WATCHMAN implantation were more frequent early in the peri-procedural period and decrease in frequency with operator experience [5]. DAT is a known complication after implant and can occur early on or late after device implant. Data on DAT is limited to initial trials and case reports.

In the PROTECT AF, the DAT incidence was 4.2%. A systemic review of 30 studies by Lempereur et al. found an overall incidence of DAT to be 3.9% (82 DAT for 2118 implanted devices) [6]. Kubo et al. reviewed 119 consecutive patients in a single center who underwent successful implantation of the WATCHMAN device. Only four patients (3.4%) developed device thrombus. All four patients had interrupted at least part of the OAC or antiplatelet regimen recommended in the PROTECT AF and PREVAIL trials. Thrombus resolved in all four patients after treatment [7]. The risk factors for DAT include higher CHA2DS2-VASc score, incomplete closure of LAA, residual flow around the device, bigger device size, interruption of recommended AC/antiplatelet protocol, lower left ventricular ejection fraction, high thrombocyte count, and patients with permanent AF [8]. The incidence of thrombus formation is assumed to be higher in the first few weeks after implant, when complete endothelialization of the device has not occurred; hence, there is a need for treatment with warfarin for at least 45 days after device implant. Treatment modalities for DAT used in the past included low molecular weight heparin (LMWH), warfarin, and new oral anticoagulants (NOACs). Lam et al. reported a case of a patient who developed DAT three years after WATCHMAN implantation due to the incomplete closure of the LAA and was successfully treated with warfarin [9].

Recurrent thrombus after successful treatment is rare. A case report by Prosperi-Porta et al. reports a 77-year-old female who developed multiple thromboembolic events after LAAOD. The thrombus resolved after three months of treatment with warfarin, but she developed a second device thrombus four months after the discontinuation of warfarin. This patient was treated with ASA and Plavix post-device implant using the ASA Plavix Feasibility Study (ASAP) strategy [10].

Our patient was started on low-dose Pradaxa (75 mg twice daily) following the device implant and developed a thrombus while taking Pradaxa, though she was compliant throughout. The thrombus resolved completely after treatment with warfarin. Pradaxa was initially started in this patient due to recent LGIB on warfarin. After the complete resolution of the first thrombus, it was deemed reasonable to stop AC and continue to observe the patient. Because she developed PE after warfarin was stopped and subsequently developed a second DAT, there were concerns she may have a hereditary hypercoagulable state. Workup was negative for protein C/S and antithrombin deficiencies, factor V Leiden, and prothrombin gene mutation. Because of the multiple thrombotic events, she was deemed a very high risk for recurrent thrombus formation, and she agreed to restart long-term anticoagulation. Eliquis was restarted and no further bleeding events have occurred.

Table 1 shows the different case reports and observational trials with details regarding when DAT occurred, the treatment used, and the outcome in seven different patients with DAT.

**Table 1:** Different case reports (1-3) and observational trials (4-7) showing when DAT occurred, the treatment used, and the outcome in seven different patients with DAT. F: female; M: male; DAT: device-associated thrombus

Author	Patient's age/sex		Treatment post-device implant	Time of diagnosis	Choice of DAT treatment	Duration of treatment	Outcome post-treatment
Kubo et al. [7]		71/F	Warfarin	45 days	Warfarin	Not mentioned	Complete resolution
Kubo et al. [7]		56/M	Warfarin	6 months	Warfarin	Not mentioned	Complete resolution
Kubo et al. [7]		61/F	Warfarin	12 months	Warfarin	Not mentioned	Complete resolution
Kubo et al. [7]		78/F	Warfarin	45 days	Warfarin	Not mentioned	Complete resolution
Wong et al. [8]		78/M	ASA+Plavix	3 years post-implant	Apixaban	3 months	Complete resolution
Lam et al. [9]		86/M	Warfarin	6 months	Warfarin	3 months	Complete resolution
Prosperi-Porta et al. [10]		77/F	ASA+Plavix+warfarin	2 months	Warfarin	6 months	Complete resolution but DAT 4 months after warfarin discontinuation

## Conclusions

DAT remains a concern after LAAOD implant. Our case report emphasizes the importance of a standardized protocol for anticoagulation post-device implant. Our patient developed DAT despite taking Pradaxa and recurrence after successful treatment with warfarin. Further studies are needed to examine the use of NOACs post-device implant and also to determine the optimal treatment for DAT. It may be reasonable to continue echocardiographic monitoring in patients who developed DAT to ensure timely diagnosis and treatment for recurrent thrombus. The ideal duration of follow-up and monitoring in cases with recurrent DAT is yet to be determined.
